# Materials and Techniques in Dentistry, Oral Surgery and Orthodontics (Second Edition)

**DOI:** 10.3390/ma19102148

**Published:** 2026-05-20

**Authors:** Maria Francesca Sfondrini, Andrea Scribante

**Affiliations:** Unit of Orthodontics and Pediatric Dentistry, Section of Dentistry, Department of Clinical, Surgical, Diagnostic and Pediatric Sciences, University of Pavia, 27100 Pavia, Italy; francesca.sfondrini@unipv.it

The field of modern dentistry is fundamentally intertwined with the continuous evolution and refinement of biomaterials. The importance of studying and developing dental materials cannot be overstated, as every clinical procedure—from the most routine restorative intervention to the most complex maxillofacial reconstruction—relies heavily on the physical, chemical, mechanical, and biological properties of the materials employed [1,2,3]. Over the past few decades, the dental profession has undergone a profound paradigm shift. It has transitioned from merely substituting missing or damaged tissues with inert, passive materials to utilizing bioactive and smart compounds that actively interact with the biological environment to promote tissue regeneration, healing, and long-term stability. This transformative journey underscores the critical need for continuous, rigorous scientific research in dental materials science [4,5,6,7,8].

The oral cavity presents a uniquely hostile environment characterized by a complex interplay of extreme conditions. Dental materials are constantly subjected to drastic fluctuations in temperature, varying pH levels from dietary intake and bacterial metabolism, complex microbiological biofilms [9,10,11,12,13,14], and intense dynamic mechanical loading generated by masticatory forces. Consequently, any material placed in the mouth must possess extraordinary mechanical resilience, chemical stability, and profound biocompatibility. The biological interface between synthetic materials and human tissues is a critical frontier. A thorough understanding of how materials interact with the dental pulp, periodontal ligament, alveolar bone, and oral mucosa is essential to prevent adverse immune responses, cytotoxicity, and premature failure of treatments [15,16,17,18,19,20,21,22].

Moreover, the digital revolution in dentistry—driven by Computer-Aided Design and Computer-Aided Manufacturing (CAD/CAM) and additive manufacturing (3D printing)—has catalyzed an unprecedented influx of novel polymers, resins, and metallic alloys into clinical practice [23,24,25,26,27]. While these technologies offer unparalleled precision and customization, they also demand rigorous scientific validation. The printing parameters, post-processing protocols, and the fundamental chemistry of these new materials must be meticulously analyzed to ensure they meet or exceed the performance of traditional counterparts [28,29,30,31,32].

Furthermore, the rise in minimally invasive dentistry has heavily relied on the advancement of adhesive technologies and biomimetic restorative materials [33,34,35,36,37]. The ability to bond predictably to enamel and dentin has allowed clinicians to preserve maximum healthy tooth structure, drastically altering treatment philosophies. However, the degradation of the hybrid layer over time and the challenge of bonding to altered dentin remain significant hurdles that ongoing research strives to overcome [38,39,40,41,42].

In this dynamic landscape, translational research serves as the vital bridge between in vitro laboratory discoveries and in vivo clinical applications. Without a deep, evidence-based understanding of material properties and limitations, clinical outcomes remain unpredictable. The primary objective of this Special Issue, “Materials and Techniques in Dentistry, Oral Surgery and Orthodontics,” is to bring together high-quality, multidisciplinary contributions that explore the cutting edge of dental material research. By investigating the latest innovations—from implant surface modifications to advanced orthodontic biomechanics—this collection aims to equip the scientific and clinical communities with the knowledge necessary to improve diagnostic accuracy, refine treatment planning, and ultimately elevate the standard of patient care.

This Special Issue presents a carefully curated collection of articles that address a multitude of current challenges within modern dental practice, highlighting the diverse and multidisciplinary nature of contemporary dental research. To provide a concise overview, these contributions can be grouped into key areas: implantology and surgery, orthodontic innovations, 3D printing technologies, preventive strategies, and comprehensive literature reviews.

In the realm of oral surgery and implantology, achieving rapid and stable osseointegration remains a primary objective. Ettuthaiyil Sambasivan et al. explored the application of organic acid-based anodization processes to produce bioactive oxides on titanium implants. Their work aims to enhance the surface topography and chemistry of implants to favor cellular attachment and biological integration. Complementing this goal, Cho et al. conducted a profound assessment of low-dose recombinant human bone morphogenetic protein-2 (rhBMP-2) combined with vacuum plasma treatments on titanium implants. This research highlights promising biological strategies to accelerate osseointegration and stimulate local bone regeneration. Furthermore, considering the surgical phase, Tsunoda et al. investigated the effect of shavings generated from 3D-printed patient-specific cutting guide materials on bone healing during jaw resection. Their findings provide vital safety insights regarding the biocompatibility of intraoperative surgical debris.

In the field of orthodontics, the demand for aesthetics and efficient mechanics continues to drive material innovation. Addressing the widespread use of clear aligners, Barbaro et al. evaluated the molecular biocompatibility of Polyethylene Terephthalate Glycol (PETG) aligners after being processed by different methods, specifically comparing laser cutting to conventional milling. This evaluation is crucial for ensuring the biological safety of the aligner edges contacting the gingival tissues. Regarding skeletal anchorage, Sfondrini et al. investigated the mechanical reliability of orthodontic miniscrews by testing the effect of collar diameter and simulated aging on their orthogonal load resistance, providing practical data for minimizing clinical failure rates.

The integration of 3D printing in dentistry was further explored in another in vitro study by Sfondrini et al., which analyzed the influence of printing orientation on the flexural strength of different light-cured resins manufactured with two distinct 3D printers. This study underscores how manufacturing parameters directly dictate the final mechanical properties of dental appliances.

Preventive and restorative dentistry are also prominently featured. Karpiński et al. evaluated the efficacy of a novel nano-hydroxyapatite-based mouthwash. Their study demonstrated its substantial activity against bacterial and fungal pathogens, alongside notable antioxidant and anti-inflammatory actions, proposing a comprehensive tool for daily oral care. In addition, Litzler et al. focused on early caries management by evaluating near-infrared transparent sealants for occlusal sealing. This in vitro study introduces promising materials that not only protect vulnerable fissures but also allow for continued diagnostic monitoring of the underlying enamel.

Finally, the issue is enriched by comprehensive reviews that synthesize current evidence to guide future clinical practices. Virvescu et al. provided a detailed update regarding the use of contemporary dental materials in periodontal regeneration, analyzing the latest scaffolds, bone grafts, and biologics used to restore the periodontium. Concurrently, Błaszczyk-Pośpiech et al. delivered a thorough current review on endodontic sealers and the latest innovations aimed at enhancing their physical, biological, and antimicrobial properties for improved root canal therapies.

Concerning future perspectives, the continuous evolution of dental materials requires a dedicated focus on developing and testing novel compounds that can better mimic natural tooth structure and biomechanics [43,44,45,46,47,48]. Future studies must heavily investigate biomimetic compounds, which hold immense potential in restorative and prosthetic dentistry due to their ability to replicate the aesthetic, physical, and mechanical properties of natural enamel and dentin [49,50,51,52,53,54]. Concurrently, the rapidly growing demand for aesthetic orthodontic treatments underscores the necessity for advanced research into materials of brackets and aligners [55,56,57,58]. Next-generation devices could incorporate shape-memory polymers and smart materials that can deliver constant, controlled forces over extended periods, thereby improving the predictability and efficiency of tooth movement [59,60,61,62,63,64]. Furthermore, significant advancements are required in the field of dental adhesives [65,66,67,68,69]. Future research should prioritize the formulation of adhesives that offer superior shear bond strength to withstand masticatory forces, while simultaneously allowing for safe debonding procedures that minimize enamel surface damage [70,71,72,73,74].

Another critical frontier in dental research is the development of innovative local drug delivery systems, particularly those involving the release of microparticles. The incorporation of antimicrobial and anti-inflammatory microparticles directly into dental materials or orthodontic adhesives could revolutionize the management of periodontal health and prevent plaque accumulation during treatment [75,76,77,78]. Moreover, a paramount concern in modern orthodontics remains the prevention and management of white spot lesions. Therefore, it is imperative to explore the use of remineralizing agents combined directly with orthodontic therapies [79,80,81,82]. Future clinical trials should focus on integrating bio-interactive remineralizing compounds—such as bioactive glass, hydroxyapatite nanoparticles, or calcium phosphate derivatives—into orthodontic cements, varnishes, and elastomeric ligatures [83,84,85,86,87]. This synergistic approach would not only correct malocclusions but simultaneously actively protect and restore the enamel structure, leading to smarter, patient-friendly therapeutic modalities.

## Data Availability

Not applicable.
